# Heavy metals, biomarkers of oxidative stress and changes in sperm function: A case-control study

**DOI:** 10.18502/ijrm.v17i3.4515

**Published:** 2019-05-29

**Authors:** Augusta Chinyere Nsonwu-Anyanwu, Eworo Raymond Ekong, Sunday Jeremiah Offor, Ogar Francis Awusha, Oliver Chukwuma Orji, Ediang Idiongo Umoh, Jennifer Aleruchim Owhorji, Faith Rowland Emetonjor, Chinyere Adanna Opara Usoro

**Affiliations:** ^1^Department of Medical Laboratory Science, Faculty of Allied Medical Sciences, College of Medical Sciences, University of Calabar, Nigeria.; ^2^Department of Medical Laboratory Science, Chemical Pathology Unit, University of Nigeria, Enugu Campus, Enugu State, Nigeria.

**Keywords:** *Heavy metals*, * Antioxidants*, * Lipid peroxidation*, * Oxidative stress*, * Sperm function.*

## Abstract

**Background:**

Heavy metal-induced oxidative stress has been implicated in abnormal sperm functions and male infertility.

**Objective:**

Serum and seminal levels of heavy metals and biomarkers of oxidative stress were compared in fertile and infertile men.

**Materials and Methods:**

A total of 130 men aged 20–60 yr comprising 30 azoospermic, 50 oligozoospermic, and 50 normozoospermic men were studied. Semen analysis was done by world health organization guidelines, biomarkers of oxidative stress (total antioxidant capacity (TAC), total plasma peroxidase (TPP), oxidative stress index (OSI), vitamin C (vit C) and nitric oxide (NO)) and fructose by colorimetry and serum and seminal heavy metals (zinc (Zn), selenium (Se), cadmium (Cd) and lead (Pb)) by atomic absorption spectrophotometry.

**Results:**

Azoospermic and oligozoospermic men had higher serum and seminal peroxides (TPP, p = 0.00), higher serum heavy metals (Zn, Se, Pb, and Cd (p = 0.01)) and lower sperm concentration, %motility, serum and seminal antioxidants (vit C, TAC, NO, GSH (p = 0.01)) compared to normozoospermic men. Higher seminal peroxides (TPP, p = 0.001), heavy metals (Pb and Cd (p = 0.03)) and lower sperm concentration, %motility, and seminal antioxidants (TAC and NO (p = 0.00)) were also observed in azoospermic men compared to oligozoospermic men. Negative correlations were observed between seminal fructose and seminal vit C (r = -0.535, p = 0.015), GSH (r = -0.734, p = 0.000), NO (r = -0.714, p = 0.000), Zn (r = -0.774, p = 0.000) and Se (r = -0.719, p = 0.000) only in azoospermic men.

**Conclusion:**

Elevated heavy metal levels, increased lipid peroxidation and antioxidant depletion is associated with abnormal sperm functions in men studied.

## 1. Introduction

Male factor infertility is increasing in developing countries and has been shown to be responsible for 40–50% of all cases of infertility in Nigeria with regional variations in prevalence and causes. Genetic, anatomical, endocrinological, immunological, radiation, infection and unexplained factors has been implicated in the etiology of male infertility (1, 2). Male infertility irrespective of etiopathologic mechanism most often presents in most cases by abnormal semen parameters (3). Toxic metals as lead and cadmium that act as endocrine disruptors have been shown to affect hormones responsible for sperm production. Induction of oxidative stress (OS) has been described as the major mechanism of heavy metal-mediated deterioration in spermatogenesis and sperm functions (4). OS, which describes a state of over abundance of reactive oxygen species (ROS) over antioxidants has been implicated in idiopathic male infertility (5). The abundance of membrane polyunsaturated fatty acid (PUFA) in spermatozoa and their capacity to generate ROS makes the spermatozoa highly susceptible to OS (3). Normal sperm functions as capacitation, motility, acrosome reaction, oocyte fusion and fertilization requires low level of ROS, however, excessive ROS is detrimental to normal sperm function by inducing peroxidation of PUFA and oxidative DNA strand breaks leading to impaired sperm viability, motility, capacitation and acrosome reaction (6). To counteract the deleterious effects of ROS, enzymatic antioxidants like catalase and superoxide dismutase (SOD) as well as non-enzymatic compounds like glutathione (GSH), vitamins C and E, zinc and selenium are present in the seminal plasma to neutralize ROS and preserve sperm function (7). Several studies have demonstrated higher levels of oxidants and heavy metals and depressed enzymatic and non-enzymatic antioxidants and essential antioxidant micronutrients in infertile men (3, 7, 8). Regional variations still exist on levels of OS indices in individuals with reproductive failure. This disparity may be attributed to the influence of infection, genetic, environmental and life style factors on oxidant/antioxidant balance. Although an association between oxidative damage and compromised sperm functions has been established, men are rarely screened for redox imbalance and treated for this condition during fertility investigations. More so, the relative or absolute contribution of OS to male factor infertility is still uncertain.

We therefore assessed the serum and seminal levels of some heavy metals, antioxidants, and biomarkers of OS and their relationship with sperm functions in fertile and infertile men.

## 2. Materials and Methods

### Study design

This case-control study was carried out at the Microbiology unit of the University of Calabar Teaching Hospital, Calabar, Nigeria from February to July 2015. Test subjects were infertile adult males (infertility for a duration of at least one year) visiting the fertility clinic for investigation for infertility. Control subjects were apparently healthy individual who had optimal sperm function after a carefully performed seminal fluid analysis and had had at least one child within the last 2 yr. The standard guidelines of the National Health Service evaluation criteria were adopted for evaluation of infertility (9). Participants with previous history of testicular surgery, genital infections, testicular varicocele, chronic organ or systemic illness, heavy smokers and chronic alcoholics were excluded from the study. In this study, infertility was defined as the inability of a couple to conceive after a period of 12 months of unprotected sexual intercourse (10), while evidence of fertility was taken as the ability to have at least one child with the last child born within the last 2 yr (10).

### Selection of subjects

The study population comprised of 130 male subjects aged 20–60 yr who fulfilled the inclusion criteria were recruited for the study after semen analysis. Anthropometric data as height, weight, hip and waist circumference were obtained and used to calculate body mass index and waist-to-hip ratio respectively. Data on socio-demographic characteristics of the subjects including family history, medical history, medications and social habits were obtained using a semi-structured questionnaire. Based on semen analysis, subjects were categorized as: Azoospermic (males with no spermatozoa in semen, n = 30), Oligozoospermic (males with a sperm concentration less than 20 million/ml, n = 50), and Normozoospermic (males with a sperm concentration greater than 20 million/ml, n = 50).

### Sample collection

#### Seminal fluid samples

Subjects were educated on the procedure for collection of semen samples. Semen samples were obtained through masturbation after 3–5 days of sexual abstinence and kept in sterile nontoxic containers, the time interval between collection and arrival to the laboratory was limited (within 30 min). The time of production, time received, and the time of analysis were recorded. Two sets of semen samples were collected from each subject of the study population within one month. The semen samples were allowed to liquefy in the incubator directly at 37${}^{\circ }$C and semen analysis was done to determine sperm volume, concentration, pH, viscocity, morphology, motility and viability as outlined in the Word Health Organisation manual for semen analysis (11). After the initial analysis, the semen samples were then centrifuged at 500 g for 10 min to separate the cells from the seminal plasma. The seminal plasma was then transferred to a clean, dry plain container and stored at –80${}^{\circ }$C until the time of analysis for seminal fructose, vit C, GSH, NO, TAC, TPP, zinc Zn, Se, Cd, and Pb.

#### Whole blood samples

A sample of 5 ml venous whole blood was collected from all subjects of the study into plain anticoagulant-free sample containers, allowed to clot and retract and then centrifuged at 500 g for 10 min at room temperature. Serum samples were collected and stored at –20${}^{\circ }$C for laboratory estimation of reduced GSH, NO, TAC, TPP, Zn, Se, Cd, and Pb.

### Laboratory methods

#### Semen analysis

####  2.4.1.1. Macroscopic evaluations

Maroscopic evaluation of the semen samples was done by determining the volume, pH, and viscosity of the sample. A glass rod was introduced into the semen sample and observation of the nature of the thread that forms on withdrawal of the rod from the sample is an estimate of the viscosity. Threads from normal semen samples should not exceed 2cm in length (12).

####  2.4.1.2. Microscopic evaluation

Wet preparation: The sample was well mixed and a drop was placed on a clean grease-free slide using a plastic Pasteur pipette and covered with a cover slip. The slide was then viewed microscopically using ×40 objective lens with the iris condenser closed to give a better contrast. The fields were examined for the presence of pus cells, epithelial cells, red blood cells, while morphology, motility, and viability of cells were determined. The morphology of the sperm cells was determined after incubating the sample with trypsin for 10 min at 25${}^{\circ }$C according to the methylene blue eosin staining procedure, feathering and fixation by flame. Using a magnification of x 1000, at least hundred sperm cells were examined. The percentage of motile spermatozoa and their mean velocity is taken as the total motility of the sperm cells.

####  2.4.1.3. Sperm concentration

This was performed using a Neubauer counting chamber. A 1:20 dilution of the sample was made using the semen-diluting fluid. The Neubauer counting chamber was then charged with the diluted semen sample and allowed to stand for 5 min before counting microscopically.

####  2.4.1.4. Estimation of heavy metals by atomic absorption spectrophotometry

Atomic Absorption spectrometry (AAS) is based on the principle that a ground state atom is capable of absorbing light of the same characteristic wavelength as it would emit if excited to a higher energy level. In flame AAS, a cloud of ground state atoms is formed by aspirating a solution of the sample into a flame of a temperature sufficient to convert the element to its atomic state. The degree of absorption of characteristic radiation produced by a suitable source will be proportional to the population of ground state atoms in the flame, and hence to the concentration of the element in the analyte (13).

####  2.4.1.5. Determination of TAC

TAC is determined based on the reaction of hydrogen peroxide (H${}_{2}$O${}_{2}$) with ferric ion-ethylenediamine tetraacetic (Fe-EDTA) complex to form hydroxyl radicals (OH). These ROS degrade benzoate leading to the release of TBARS (thiobarbituric acid reactive substance). The production of TBARS is suppressed by antioxidants present in the sample and this inhibition of colour development is termed the total antioxidant of the sample measured spectrophotometrically at 532 nm (14).

####  2.4.1.6. Estimation of TPP

TPP is determined based on the ferrous-butylated hydroxytoluene-xylenol orange complex (FOX-2) test system which is based on the oxidation of ferrous ions to ferric ions by various types of peroxides present in the serum sample; to produce a colored ferric-xylenol orange complex whose absorbance is measured spectrophotometrically at 560nm (15).

####  2.4.1.7. Calculation of oxidative stress index (OSI)

The ratio of TPP to TAC was calculated as the OSI, an indicator of the degree of OS.

$$\mathrm{OSI}\left(\%\right)=\frac{[TPP\left(\mu mol/H_{2}O_{2}\right]\times 100}{\left[TAC(\mu mol)\right]}$$

####  2.4.1.8. Estimation of NO

The Griess test was used for detecting the total levels of nitrite or nitrous acid in samples. The NO-containing compounds in the serum combines with alpha-Naphthylamine to produce pink azo dye whose absorbance was measured at a wavelength of 540 nm. The measurement of nitric oxide metabolites which is estimated as total nitrite and nitrate levels, is considered as a direct marker of invivo NO production (16).

####  2.4.1.9. Estimation of GSH

Estimation of GSH was carried out following the modified standard Ellman's method. The GSH in the sample reacts with Ellman's reagent (5-5'-dithiobis-2-nitrobenzoic acid (DNTB)) to form the chromophore 5-thionitrobenzoic acid (TNB) and GS-TNB whose absorbance is measured at 412 nm (17).

####  2.4.1.10. Estimation of fructose

In the presence hydrochloric acid (HCl) under heat, fructose reacts with indole to produce a colored complex whose absorbance is measured at wavelength of 450–492 nm (18).

####  2.4.1.11. Estimation of vit C using the modified reduction method

Ascorbic acid is converted to dehydro-ascorbic acid by shaking with Norit-activated charcoal, and this is then coupled with 2,4-dinitrophenyl hydrazine in the presence of thiourea as a mild reducing agent. In the presence of sulphuric acid, dinitrophenyl hydrazone which is a red compound is assayed colorimetrically. The absorbance of colored compound is proportional to vit C concentration in the sample (19).

####  2.4.1.12. Ethical consideration

This study was carried out in accordance with the Ethical Principles for Medical Research Involving Human Subjects as outlined in the Helsinki Declaration in 1975 and subsequent revisions. Informed consent was obtained from the subjects before recruitment into the study.

####  2.4.1.13. Statistical analysis

Results were presented as mean ± standard deviation. Data was analysed using the statistical package for social sciences (SPSS version 20.0, IBM, USA). One way analysis of variance (ANOVA) was used to test the variations within and among group means and Fisher's least significant difference (LSD) post-hoc test was used for the comparison of multiple group means. Pearson's correlation was used to determine the associations between variables. A probability value p < 0.05 was considered statistically significant.

## 3. Results

The age, body mass index (BMI), semen parameters, fructose, seminal OS indices and heavy metals in normozoospermic, oligozoospermic and azoospermic males are presented in Table I. Significant variations were observed in semen volume, sperm concentration and seminal fructose, vit C, GSH, NO, TAC, TPP, OSI, Cd and Pb levels among normozoospermic, oligozoospermic and azoospermic men studied (p = 0.000). No significant variations were observed in the mean age, BMI, seminal GSH, Zn, Se, Pb and Cd among the three groups (p = 0.051). Table II shows the comparison of semen volume, sperm concentration and seminal fructose, vit C, GSH, NO, TAC, TPP, OSI, Cd and Pb levels among normozoospermic, oligozoospermic and azoospermic men using LSD post hoc analysis. Normozoospermic men had significantly higher semen volume and sperm concentration, seminal fructose, vit C, TAC, NO and lower seminal TPP and OSI compared to azoospermic men; and higher %motility, sperm count, seminal fructose, vit C, TAC and lower seminal TPP compared to oligozoospermic men (p = 0.026). Oligozoospermic men had higher sperm concentration, seminal TAC, NO, TPP, Cd and Pb and lower seminal OSI compared to azoospermic men (0.032). Table III shows serum OS indices and heavy metals in normozoospermic, oligozoospermic and azoospermic men studied. Significant variations were observed in the serum levels of GSH, NO, TAC, TPP, OSI, Zn, Se, Pb and Cd among normozoospermic, oligozoospermic and azoospermic men studied (0.018). Comparison of serum GSH, NO, TAC, TPP, OSI, Pb and Cd in normozoospermic, oligozoospermic, and azoospermic men using LSD post hoc are presented in Table IV. Normozoospermic men had significantly higher serum GSH, NO and TAC and lower TPP, OSI, Zn, Pb and Cd compared to oligozoospermic and azoospermic men studied (0.26). Table V shows the correlation of seminal fructose against seminal vit C, GHS, NO, Zn and Se in azoospermic men. Significant negative correlations were observed between seminal fructose and seminal vit C (r = -0.535, p = 0.015), GSH (r = -0.734, p = 0.000), NO (r = 0.714, p = 0.000), Zn (r = 0.774, p = 0.000) and Se (r = 0.719, p = 0.000) in azoospermic men.

**Table:** Age, BMI, semen parameters, fructose, seminal OS indices and heavy metals in Normozoospermic, Oligozoospermic and Azoospermic men

	**Normozoospermic (n = 50)**	**Oligozoospermic (n = 50)**	**Azoospermic(n = 30)**	**F ratio**	**p-value**
Age (yrs)	34.26 ± 4.04	33.57 ± 3.77	32.85 ± 3.22	0.91	0.406
BMI (kg/m${}^{2}$)	27.06 ± 4.76	26.12 ± 4.69	26.10 ± 4.49	0.44	0.646
S Volume (ml)	4.29 ± 1.615	3.63 ± 1.57	2.13 ± 1.20	12.98	0.000*
S Concentration (10${}^{6}$)	78.27 ± 31.41	8.09 ± 5.52	0.00 ± 0.00	144.89	0.000*
S fructose (µmol/ml)	13.71 ± 1.71	9.74 ± 2.16	9.51 ± 1.53	50.41	0.000*
S Vit C (mg/dl)	2.26 ± 0.81	1.46 ± 0.78	1.35 ± 0.58	13.20	0.000*
S TPP (µmolH${}_{2}$O${}_{2}$/L)	336.53 ± 70.37	386.47 ± 87.42	472.01 ± 128.81	13.69	0.000*
S TAC (µmol/l)	1915.74 ± 734.55	1183.33 ± 192.31	679.29 ± 121.33	46.31	0.000*
S OSI (%)	26.18 ± 24.54	33.35 ± 8.25	69.46 ± 11.68	43.56	0.000*
S GSH (µmol/l)	8.46 ± 2.76	8.06 ± 2.54	6.93 ± 5.79	1.18	0.311
S NO (µmol/l)	4.56 ± 1.15	4.25 ± 0.83	2.37 ± 0.78	36.09	0.000*
S Zn (µg/L)	100.53 ± 16.85	104.56 ± 15.94	100.310 ± 3.14	0.85	0.430
S Se (µg/L)	26.74 ± 22.93	23.81 ± 12.75	20.88 ± 9.98	0.77	0.465
S Pb (µg/L)	0.47 ± 0.17	0.53 ± 0.17	0.43 ± 0.15	2.57	0.083
S Cd (µg/L)	0.41 ± 0.15	0.47 ± 0.16	0.37 ± 0.13	3.08	0.051
Note: Data presented as mean ± SD; *= significant at p < 0.05, using analysis of variance					

BMI = Body mass index	Vit C = Vitamin C	TPP = Total plasma peroxides
TAC = Total antioxidant capacity	OSI = Oxidative stress index	GSH = Glutathione
NO = Nitric oxide	Zn = Zinc	Se = Selenium
Pb = Lead	Cd = Cadmium	S = Seminal

**Table:** Comparison of sperm volume, motility, count and seminal fructose, vitamin C, GSH, NO, TAC, TPP, OSI, Cd and Pb levels among Normozoospermic, Oligozoospermic and Azoospermic men using LSD post hoc analysis

**Parameter**	**Groups**		**Mean diff.**	**p-value**
	**Normozoospermic n = 50**	**Azoospermic n = 30**	
S Volume (ml)	4.29 ± 1.615	2.13 ± 1.20	2.16 ± 0.443	0.000*
S Concentration (10${}^{6}$)	78.27 ± 31.41	0.00 ± 0.00	78.27 ± 5.58	0.000*
S Fructose (µmol/ml)	13.71 ± 1.71	9.51 ± 1.53	4.19 ± 0.52	0.000*
S Vit C (mg/dl)	2.26 ± 0.81	1.35 ± 0.58	0.90 ± 0.21	0.000*
S TPP (µmolH${}_{2}$O${}_{2}$/L)	336.53 ± 70.37	472.01 ± 128.81	-35.48 ± 25.91	0.000*
S TAC (µmol/L)	1915.74 ± 734.55	679.29 ± 121.33	1236.45 ± 134.00	0.000*
S OSI (%)	26.18 ± 24.54	69.46 ± 11.68	-43.28 ± 4.78	0.000*
S NO (µmol/L)	4.56 ± 1.15	2.37 ± 0.78	-78.27 ± 5.58	0.000*
	**Normozoospermic n = 50**	**Oligozoospermic n = 50**	
S Motility (%)	73.14 ± 6.65	36.43 ± 19.27	36.71 ± 3.05	0.000*
S Concentration (10${}^{6}$)	78.27 ± 31.41	8.09 ± 5.52	70.17 ± 4.77	0.000*
S Fructose (µmol/ml)	13.71 ± 1.71	9.74 ± 2.16	3.96 ± 0.45	0.000*
S Vit C (mg/dl)	2.26 ± 0.81	1.46 ± 0.78	0.79 ± 0.18	0.000*
S TPP (µmolH${}_{2}$O${}_{2}$/L)	336.53 ± 70.37	386.47 ± 87.42	-49.94 ± 22.09	0.026*
S TAC (µmol/L)	1915.74 ± 734.55	1183.33 ± 192.31	732.41 ± 114.27	0.000*
	**Azoospermicn = 30**	**Oligozoospermic n = 50**	
S Volume (ml)	2.13 ± 1.20	3.63 ± 1.57	-1.50 ± 0.43	0.001*
S TPP (µmolH${}_{2}$O${}_{2}$/L)	472.01 ± 128.81	386.47 ± 87.42	85.54 ± 25.91	0.001*
S TAC (µmol/L)	679.29 ± 121.33	1183.33 ± 192.31	-504.04 ± 134.00	0.000*
S OSI (%)	69.46 ± 11.68	33.35 ± 8.25	36.12 ± 4.79	0.000*
S NO (µmol/L)	2.37 ± 0.78	4.25 ± 0.83	-1.89 ± 0.27	0.000*
S Pb (µg/L)	0.43 ± 0.15	0.53 ± 0.17	-0.10 ± 0.05	0.032*
S Cd (µg/L)	0.37 ± 0.13	0.47 ± 0.16	-0.09 ± 0.04	0.021*
Note: Data presented as mean ± SD; *= significant at p < 0.05				

Vit C = Vitamin C	TPP = Total plasma peroxides
TAC = Total antioxidant capacity	OSI = Oxidative stress index
NO = Nitric oxide	Pb = Lead
Cd = Cadmium	S = Seminal
**Se & Zn were not included here because their values were not significantly different between the groups.	

**Table:** Serum OS indices and heavy metals in Normozoospermic, Oligozoospermic and Azoospermic males

**Index**	**Normozoospermic** ** (n = 50)**	**Oligozoospermic** ** (n = 50)**	**Azoospermic** ** (n = 30)**	**F ratio**	**p-value**
P TPP (µmolH${}_{2}$O${}_{2}$/L)	100.36 ± 8.52	118.69 ± 30.43	130.47 ± 23.27	12.489	0.000*
P TAC (µmol/L)	1501.13 ± 333.10	1242.93 ± 285.00	1140.21 ± 343.99	9.919	0.000*
P OSI (%)	7.12 ± 2.21	10.18 ± 3.96	12.43 ± 4.21	16.214	0.000*
P GSH (µmol/l)	16.39 ± 6.19	11.29 ± 4.32	10.63 ± 1.48	13.555	0.000*
P NO (µmol/L)	2.28 ± 0.47	1.99 ± 0.21	1.93 ± 0.36	8.038	0.001*
P Zn (µg/L)	108.04 ± 0.94	168.23 ± 0.47	155.22 ± 0.37	7.269	0.001*
P Se (µg/L)	92.39 ± 9.06	115.62 ± 6.28	107.18 ± 5.74	2.653	0.076
P Pb (µg/L)	0.46 ± 0.40	0.72 ± 0.20	0.66 ± 0.16	7.282	0.001*
P Cd (µg/L)	0.219 ± 14.43	0.287 ± 10.31	0.305 ± 9.61	4.216	0.018*
Note: Data presented as mean ± SD; *= significant at p < 0.05, using analysis of variance					

p = Serum	TPP = Total plasma peroxides	TAC = Total antioxidant capacity
OSI = Oxidative stress index	GSH = Glutathione	NO = Nitric oxide
Zn = Zinc	Se = Selenium	Pb = Lead
Cd = Cadmium	

**Table:** Comparison of serum GSH, NO, TAC, TPP, OSI, Pb and Cd in Normozoospermic, Oligozoospermic and Azoospermic men using LSD post hoc analysis

**Parameter**	**Groups**		**Mean diff.**	**p-value**
	**Normozoospermicn = 50**	**Azoospermicn = 30**	
P TPP (µmolH${}_{2}$O${}_{2}$/L)	100.36 ± 8.52	130.47 ± 23.27	-30.11 ± 6.32	0.000*
P TAC (µmol/L)	1501.13 ± 333.10	1140.21 ± 343.99	360.92 ± 89.06	0.000*
P OSI (%)	7.12 ± 2.21	12.43 ± 4.21	-5.31 ± 0.96	0.000*
P GSH (µmol/L)	16.39 ± 6.19	10.63 ± 1.48	5.77 ± 1.34	0.000*
P NO (µmol/L)	2.28 ± 0.47	1.93 ± 0.36	0.35 ± 0.10	0.001*
P Zn (µg/L)	108.04 ± 0.94	155.22 ± 0.37	-46.18 ± 0.19	0.017*
P Pb (µg/L)	0.46 ± 0.40	0.66 ± 0.16	-0.19 ± 0.08	0.017*
P Cd (µg/L)	0.22 ± 14.43	0.30 ± 9.61	-0.08 ± 3.35	0.013*
	**Normozoospermicn = 50**	**Oligozoospermic n = 50**	
P TPP (µmolH${}_{2}$O${}_{2}$/L)	100.36 ± 8.52	118.69 ± 30.432	-18.33 ± 5.39	0.001*
P TAC (µmol/L)	1501.13 ± 333.10	1242.93 ± 285.00	258.20 ± 75.95	0.001*
P OSI (%)	7.12 ± 2.21	10.18 ± 3.96	-3.05 ± 0.83	0.000
P GSH (µmol/L)	16.39 ± 6.19	11.29 ± 4.32	5.10 ± 1.14	0.000*
P NO (µmol/L)	2.28 ± 0.47	1.99 ± 0.21	0.29 ± 0.09	0.001*
P Zn (µg/L)	108.04 ± 0.94	168.23 ± 0.47	-60.19 ± 0.16	0.000*
P Se (µg/L)	92.39 ± 53.62	115.62 ± 37.21	-23.23 ± 10.16	0.025*
P Pb (µg/L)	0.46 ± 0.40	0.72 ± 0.20	-0.26 ± 0.07	0.000*
P Cd (µg/L)	0.22 ± 14.43	0.29 ± 10.31	-0.067 ± 2.86	0.021*
Note: Data presented as mean ± SD; *= significant at p < 0.05				

p = Serum	TPP = Total plasma peroxides	TAC = Total antioxidant capacity
OSI = Oxidative stress index	GSH = Glutathione	NO = Nitric oxide
Zn = Zinc	Pb = Lead	Se = Selenium
Cd = Cadmium	

**Table:** Correlation of seminal fructose against seminal vitamin C, GSH, NO, Zn and Se in Azoospermic men

**Index**		**r-value**	**p-value**
sFructose (µmol/ml)	sVit. C (mg/dl)	–0.535	0.015*
	sGSH (µmol/L)	–0.734	0.000*
	sNO (µmol/L)	–0.714	0.000*
	sZn (µg/L)	–0.774	0.000*
	sSe (µg/L)	–0.719	0.000*
Note:*= Significant at p < 0.05			

S = Seminal	Vit C = Vitamin C	GSH = Glutathione
NO = Nitric oxide	Zn = Zinc	Se = Selenium

## 4. Discussion

Abnormal sperm function and male infertility have been attributed to several factors including increased generation of ROS and OS which may be related to heavy metal accumulation. We examined the association between heavy metals, OS indices and defective sperm functions in fertile and infertile men.

In this study, significantly lower serum and seminal TAC and higher TPP and OSI were observed in oligozoospermic and azoospermic men compared to their normozoospermic counterparts. The spermatozoa like any other cell under aerobic condition generate ROS whose activities are modulated by several enzymatic and non-enzymatic antioxidant systems (15). Overabundance of ROS results in sperm damage and impaired sperm functions, the extent of damage being dependent on the nature, amount, and duration of ROS insults (5, 20). Increased TPP and OSI seen in oligozoospermic and azoospermic men compared to their normozoospermic counterparts may therefore be attributed to increased generation of ROS leading to increased membrane peroxidation and depressed antioxidants observed in these groups. Higher TPP and OSI and decreased TAC was also observed in azoospermic compared to oligozoospermic men. This may be an indication that azoospermic men generate the highest levels of ROS which may contribute to the absence of spermatozoa in their semen. Infertile men have been reported to have elevated levels of ROS, high enough to induce apoptosis compared to their fertile counterparts (21). Lower sperm concentration and percentage motility were observed in oligozoospermic and azoospermic men compared to normozoospermic men studied. This may result from increased lipid peroxidation induced by ROS (higher TPP) and the consequent antioxidant depression (lower TAC) observed in these individuals. Excess ROS and OS have been shown to exert toxic effects on sperm function leading to impaired sperm motility, concentration, and morphology (5, 22). Impaired motility may result from inhibition of mitochondrial function, decreased axonemal protein phosphorylation, decreased sperm viability, increased mid-piece sperm defects, and sperm immobilization arising from lipid peroxidation; leading to reduced hyperactivation, oocyte penetration, and poor fertilization outcome. Pathological levels of ROS have been shown to cause oxidative DNA damage and apoptosis of spermatozoa resulting in cell death and reduced sperm concentration. Higher percentage of apoptotic spermatozoa have been demonstrated in oligoasthenozoospermic subjects than in normozoospermic men (4). Seminal fructose and vit C levels were significantly lower in oligozoospermic and azoospermic men compared to normozoospermic men studied. Seminal fructose, produced by seminal vesicles and the ampulla of the ductus deferens has been described as the major source of energy for spermatozoa and has been used as marker of seminal vesicle functions (23). Lower fructose levels coupled with lower ejaculate volume and sperm motility observed in oligozoospermic and azoospermic may be indicative of ejaculatory duct obstruction, seminal vesicle dysfunction, or hypoplasia. Contrary to our findings, higher seminal fructose levels often accompanied by lower sperm concentration and sperm motility have been reported (24). Negative associations were observed between seminal fructose and seminal antioxidant and antioxidant elements in azoospermic men studied. Similarly, an inverse relationship has been reported between seminal zinc and fructose concentrations. Effective seminal fluid activity has been related to the inverse relationship between seminal zinc and fructose levels (25). Association between vit C and normal functioning of the male reproductive system has been established. High concentration of vit C has been localized in the epididymal fluid and seminal plasma and has been associated with ejaculate volume, sperm count and sperm motility (26). Vit C has been described as a major antioxidant in the testis; it neutralizes ROS thereby protecting the spermatozoa from lipid peroxidation and oxidative DNA damage. Lower vit C levels seen in both oligozoospermic and azoospermic subjects may result from increase demand in ROS neutralization (26). Also lower sperm count and motility in this group may be a consequence of lower seminal vit C status. Improvement in sperm numbers, concentration, motility and amelioration of male reproductive traits with high fertility rates has been observed with vit C supplementation (27).

The serum NO levels of normozoospermic men were significantly higher than those of oligozoospermic and azoospermic men and seminal NO higher in normozoospermic compared to azoospermic men studied. NO has been described as a mediator of several physiopathological events including regulation of vascular tone, neurotransmission, apoptosis, and inflammation (28). Nitric oxide has been localized in the spermatozoa, and has been reported to influence sperm function both positively and negatively. Low NO levels have been shown to increase sperm motility, capacitation, zona pellucida sperm-binding protein, and sperm viability (22). Higher levels of NO however have been associated with defective sperm motility, morphology, sperm count and viability through mechanisms that involves the inhibition of mitochondrial respiration that results in a depletion of adenosine triphosphate and the reduction of sperm motility (29). The effects of NO on sperm functions have been reported to be a function of the alternative redox state and relative NO level (30). Therefore, higher NO observed in normozoospermic men without defective sperm functions may be related to their normal redox status as shown by their higher TAC and lower TPP levels. Azoospermic and Oligozoospermic subjects on the other hand have relatively defective sperm function irrespective of their lower levels of NO which physiologically is supposed to increase sperm motility and viability. Defective sperm functions at lower NO levels in these subjects may therefore be related to their abnormal redox status as depicted by lower TAC levels and increased lipid peroxidation (30). Lower serum GSH levels were observed in azoospermic and oligozoospermic men compared to normozoospermic men. GSH, the most abundant non-enzymatic antioxidant has been detected in high concentrations in male reproductive tissues. The ability of GSH to react directly with ROS by donating hydrogen ion and also serve as a cofactor for antioxidant enzyme glutathione peroxidase (GPX) is responsible for its scavenging properties. GSH protects the sperm plasma membrane by neutralizing the cytotoxic aldehydes produced during lipid peroxidation (31). Normal sperm function seen in normozoospermic men may therefore be an indication of the significant role played by higher GSH levels in protecting the spermatozoa against oxidative damage (7). Decreased serum GSH in oligozoospermic and azoospermic men studied may be attributed to their increased consumption to buffer the effects of increased ROS (decreased TAC) and lipid peroxidation (increased TPP) observed in this group. Disruption in the membrane integrity of spermatozoa leading to defective motility associated with OS has been linked to low seminal plasma and erythrocyte GSH levels (32). The serum levels of the antioxidant metals zinc and selenium were significantly higher in oligozoospermic and azoospermic men compared to their normozoospermic counterparts. However, no significant differences were observed in the seminal levels of these elements in normozoospermic compared to oligozoospermic and azoospermic. A similar observation has been reported by another study (33). Higher zinc and selenium levels in oligozoospermic and azoospermic compared to normozoospermic may be related to increased lipid peroxidation in these subjects. The body in response to increased lipid peroxidation may enhance the activities of the antioxidant enzymes glutathione peroxidase and Copper/Zn-SOD, which have selenium and zinc as components thereby leading to increased zinc and selenium levels observed. Contrary to our findings, higher serum and seminal levels of zinc and selenium have been demonstrated in normozoospermic subjects compared to oligozoospermic and azoospermic subjects (34, 35). Seminal Zn and Se have been shown to be essential for normal testicular development and spermatogenesis (7); Zn has been described as the primary factor responsible for DNA transcription and protein synthesis during spermatogenesis (34). It is also a component of Copper/Zn-SOD antioxidant enzyme. Reduced levels have been associated with decline of its antioxidant capacity, increased lipid peroxidation and low sperm quality. Normal sperm function and protection against oxidative DNA damage requires Se (35). Increased incidence of sperm shape abnormalities, breakage at the middle piece and loss of motility has been associated with low levels of Se (7). The serum levels of the heavy metals lead and cadmium were higher in oligospermic and azoospermic men compared to normospermic subjects studied. Oligospermic men also had higher seminal Pb and Cd compared to azoospermic men. The negative impacts of Cd and Pb on the motility, morphology, quality and concentration of spermatozoa have been described. The chemical affinity of Cd for calcium ions in the cell membranes may alter the integrity of the acrosomal membrane of the spermatozoa leading to abnormal acrosomal reaction and impaired fertility. Cd has been implicated in modulating the initiation and duration of acrosome reaction and occurrence of separated flagellum (36). Possible damaging effect of Pb on the chromatin of spermatozoa and spermatogenesis has been reported. Defective sperm function associated with lead exposure has been linked to lead-associated increase in lipid peroxidation (37). Pb has been shown to reduce normal sperm metabolism through suppression of sperm creatine kinase activity thereby contributing to infertility in men (38). Subjects recruited into this study were not occupational exposed to lead and cadmium and other confounding factors as cigarette smoking were eliminated. Alterations in the homeostasis of seminal trace element composition have been shown to influence the fertility capacity of the spermatozoa (33).

## 5. Conclusion

The findings of this study have shown that abnormal sperm functions may be related to elevated levels of heavy metals, increased lipid peroxidation and antioxidant depletion which may result in impaired fertility in men studied. Antioxidant supplementation may be beneficial in amelioration of impaired semen functions.

##  Conflict of Interest

The authors declare that there is no conflict of interests regarding the publication of this article.
